# INTERNATIONALLY EDUCATED NURSES CARING FOR OLDER ADULTS: A SCOPING REVIEW

**DOI:** 10.1093/geroni/igac059.2492

**Published:** 2022-12-20

**Authors:** Sherif Olanrewaju, Susan Loeb, Manuel Rosaldo

**Affiliations:** Penn State University, BOALSBURG, Pennsylvania, United States; The Pennsylvania State University, State College, Pennsylvania, United States; The Pennsylvania State University, State College, Pennsylvania, United States

## Abstract

Internationally Educated Nurses (IENs) are nurses who are born and obtained their licenses in their home country and relocate to work in a different country. IENs are increasing being recruited to work in Western countries to address nursing shortages. Estimates indicate that IENs account for 5-8 percent of registered nurses in the United States (US). The purpose of this scoping review was to identify and synthesize research evidence on IENs' experiences caring for older adults. A 5-step process for Scoping Reviews was applied, which includes: identifying the research question; identifying relevant studies; study selection; charting the data; and collating, summarizing, and reporting the results. A search was conducted in Web of Science, PubMed, CINAHL, PsycINFO, and Google Scholar. Keywords included but were not limited to IENs, older adults, and long-term care setting. Inclusion criteria were (1) empirical studies examining IENs providing direct care for older adults in any healthcare settings and (2) original research published in English. A total of 13 articles were selected for inclusion (nine quantitative and four qualitative studies). The studies were conducted in the US (n=10), the Netherlands (n=1), Australia (n=1), and New Zealand (n=1). Results revealed three primary themes: transitional challenges, IENs' experiences working with older adults, and factors affecting IEN capacity to deliver services. Study findings are relevant to nursing leaders and policymakers in developing culturally relevant programs to help IENs transition successfully into the nursing workforce. Additional qualitative research is required to explore lived experiences of IENs caring for older adults.

